# Response to semaglutide of non-drinker subjects with type 2 diabetes

**DOI:** 10.1186/s13098-024-01344-6

**Published:** 2024-05-17

**Authors:** Giovanni Petralli, Francesco Raggi, Alice Del Zoppo, Chiara Rovera, Antonio Salvati, Maurizia Rossana Brunetto, Anna Solini

**Affiliations:** 1 Department of Surgical Medical, Molecular and Critical Area Pathology, University of Pisa, Via Roma 67, Pisa, I-56126 Italy; 2 Hepatology Unit, Azienda Ospedaliero Universitaria Pisana, Pisa, Italy; 3 Department of Clinical and Experimental Medicine, University of Pisa, Pisa, Italy

**Keywords:** Interleukin-18, Liver stiffness, Semaglutide, Non-drinker, Type 2 diabetes

## Abstract

**Background:**

Metabolic dysfunction-associated Steatotic Liver Disease (MASLD) displays a worse prognosis in subjects with type 2 diabetes (T2D); effective treatments are, so far, scanty. Semaglutide showed efficacy in improving steatohepatitis. We longitudinally observed a MASLD cohort of T2D subjects starting semaglutide, to detect an improvement of non-invasive surrogates of steatosis and fibro-inflammatory liver involvement, evaluating the role of mild alcohol consumption.

**Patients and methods:**

In 62 overweight/obese T2D subjects with MASLD (36 non-drinker and 26 mild alcohol consumers), anthropometric, bio-humoral and transient elastography (TE) data were collected before (T0) and after an average time of 6.4 month (T1) from injective semaglutide prescription. Circulating levels of hormones (GIP, GLP-1, glucagon, insulin) and inflammatory markers (TNFα, MCP-1, IL-18, IL-10) were measured. Steatotic and necro-inflammatory liver involvement was evaluated with FibroScan controlled attenuation parameter (CAP) and liver stiffness (LS), respectively.

**Results:**

Significant (*p* < 0.006) T0-T1 reductions of BMI, waist circumference, fasting glucose, and HbA1c were observed. AST (-10 ± 3 IU/L), ALT (-18 ± 5 IU/L), GGT (-33 ± 15 IU/L), CAP (-25 ± 8 dB/m) and LS (-0.8 ± 0.4 kPa) were reduced, too. GLP-1 increased (+ 95.9 pM, *p* < 0.0001) and IL-18 was reduced (-46.6 pg/ml, *p* = 0.0002). After adjustment for confounders, CAP improving was only related to GLP-1 increase (ß=-0.437, *p* = 0.0122). Mild alcohol intake did not influence these relations.

**Conclusion:**

Use of semaglutide in subjects with T2D and MASLD is associated with a significant decline of liver steatosis and necroinflammation proxies; mild alcohol assumption did not exert any influence. An independent effect of GLP-1 raise was observed on reduction of steatosis, irrespective of alcohol consumption.

**Supplementary Information:**

The online version contains supplementary material available at 10.1186/s13098-024-01344-6.

## Introduction

Metabolic dysfunction associated Steatotic Liver Disease (MASLD), formerly Non-Alcoholic Fatty Liver Disease (NAFLD), leading cause of chronic liver disease in high-income countries, is strictly related with metabolic syndrome, being prevalent in subjects with obesity, type 2 diabetes (T2D) and dyslipidaemia [1]. Indeed, MASLD encompasses a large spectrum of conditions, ranging from simple steatosis to non-alcoholic steatohepatitis (NASH), advanced fibrosis, liver cirrhosis and hepatocellular carcinoma; such broad field of phenotypes requires the identification of early predictors of more serious clinical outcomes [2]. Liver biopsy is unanimously recognized as the gold standard for fibrosis detection and quantification in any patient with chronic liver disease, irrespective of its etiology. However, it is a costly procedure, at risk of significant complications, and it cannot be used for monitoring fibrosis evolution over time. Among various non-invasive tools developed to detect and quantify liver fibrosis, liver stiffness measured by transient elastography (FibroScan) and fibrosis-4 index (FIB-4) show a very good diagnostic performance [3, 4].

The differential diagnosis between alcoholic and non-alcoholic fatty liver disease is schematically based on the evaluation of a daily alcohol intake of 30 g for men and 20 g for women, assuming that a lower volume of alcohol consumption would not influence the occurrence of liver steatosis [5, 6]; inherited factors might also play a role, and suggestive inverse associations of genetically-predicted alcohol, coffee, and caffeine consumption, and vigorous physical activity with MASLD risk have been recently reported [7]. However, whether or not the absolute absence of alcohol consumption might help in reducing the effect of metabolic comorbidities on MASLD is still unclear [8]: some studies showed a detrimental effect in individuals with MASLD [9, 10], while others seem to suggest a possible protective effect of light or moderate alcohol consumption [11–13].

In T2D subjects treated with semaglutide, a GLP-1 analogue, robust evidence sustains better glycemic control, weight loss and improved cardiovascular outcomes [14–16]; furthermore, clinical studies show as semaglutide might reduce hepatic steatosis and inflammation [17], while data on down-staging of fibrosis are less conclusive [18, 19]. No studies have, to date, compared the effect of this molecule in non-drinker vs. moderately alcohol consuming T2D subjects, either in terms of metabolic profile, liver function and morphology, and markers of subclinical inflammation and oxidative stress; the present study has been designed to address these issues.

## Subjects and methods

### Study design

Caucasian subjects with T2D and MAFLD consecutively attending the Internal Medicine Metabolic Clinic of the University of Pisa between January and December 2023 and deserving semaglutide treatment based on clinical judgement (HbA1c ≥ 7.0 < 9.0% and high cardiovascular risk due to presence of comorbidities, or target organ damage, or any previous cardiovascular event) were enrolled on a volunteer basis in this observation. Exclusion criteria were active or recent (< 2 yrs) neoplasm, systemic inflammatory diseases, any ongoing pharmacologic treatment excluding antihypertensive and hypolipidemic drugs or metformin, denied informed consent, relevant alcohol assumption (AUDIT score > 7) [20], or absence of ultrasonographic detection of liver steatosis at a preliminary ultrasonographic evaluation.

At the baseline visit, information regarding personal medical history, medication and family history was collected, and smoking habits were recorded. BMI was calculated and waist circumference was measured in all participants; repeated sitting BP measurements according to the international standard procedure were recorded. Alcohol consumption (in g/wk, assuming 10 g as the alcohol content of a standard drink) was estimated through the AUDIT Score supplemented with images for drink size.

A blood sample was drawn from the antecubital vein, and plasma and serum aliquots were collected and frozen at -20 °C until required for quantitation); urine samples were also collected and frozen for further analyses. Weekly injective semaglutide was started and titrated up to 1 mg/week according to international indications (0.25 mg for month 1, 0.50 mg for month 2, 1 mg/week from month 3 to the follow up visit). Participants were asked to refrain from modifying their dietary habits and usual physical activity for the whole study period. All determinations were repeated six months ± 2 weeks from the baseline visit.

### Liver ultrasonography

The day before starting semaglutide, all participants underwent liver fat content (Controlled Attenuation Parameter, CAP) and stiffness (LS) measurement by Fibroscan® (EchoSens, Paris, France) in the fasting state. All measures were performed by the same expert physician (G.P.) on the liver right lobes in patients lying on their back, right arm in maximal abduction. The ultrasound (US) guide was used to identify a target liver area (≥ 6 cm thick without major vascular structures). The procedure was based on at least 10 validated measurements. LS was recorded in kilopascals as the median value of all measurements. CAP was recorded in dB/m with values range from 100 to 400 dB/m. LS value was analysed both as continuous variables and as categorical one, based on cut-offs of 8 kPa and 9.7 kPa; CAP like above, with cut-offs of 268 dB/m able to detect significant steatosis [21]. The same determination was repeated within 3 days from the 6-month follow up visit.

*Biochemistry and hormone profile* Routine blood laboratory tests (fasting glucose, lipid profile, serum creatinine) were measured by standard methods in the biochemistry laboratory of the University Hospital in Pisa. Fasting plasma and serum concentrations of GLP-1, GIP, insulin and glucagon were measured using an enzyme‑linked immunosorbent assay (GLP-1: EZGLP1T-36 K; Millipore Corporation, Billerica, MA, USA; GIP: EZHGIP-54 K, Millipore Corporation; insulin: I10-1113-10, Mercodia AB, Uppsala, Sweden; glucagon: 10-1271-01, Mercodia, respectively), in accordance with the manufacturer’s instructions. To estimate insulin sensitivity, the Homeostatic Model Assessment for Insulin Resistance (HOMA-IR) index was calculated.

### Inflammatory markers

TNF-a, MCP-1, IL-18 and IL-10 were measured by Luminex Human Discovery 4-Plex Assays (LXSAHM-04, Bio-Techne/R&D Systems, Minneapolis, MN, USA) according to the manufacturer’s procedure. Data was acquired on a Luminex FlexMap 3D analyzer (Luminex Corp., Austin, TX, USA) and analyzed by xPONENT Software 4.0 (ThermoFisher Scientific, Waltham, MA, USA) with 5PL logistic curves.

### Sample size calculation

A total sample size of 48 patients (24 subjects per group) was calculated to provide at least 80% power to detect a 5 mmol/mol difference in HbA1c reduction after semaglutide treatment, deemed clinically relevant, in a two-tailed non-parametric group comparison (α = 0.05), assuming an SD of 6 mmol/mol.

### Statistics

Data are reported as mean ± SD or median (IQ range). Paired t-student test and Wilcoxon test were performed to compare T0-T1 paired data when variable distribution was normal or not-normal, respectively. Differences were analysed by Kruskal-Wallis test for continuous variables and Fisher exact test for categorical variables, followed by post-hoc pairwise comparisons as appropriate. Variables with a skewed distribution were log-transformed before mixed model analysis. Bivariate correlations were tested using Kendall’s correlation. To account for potential sex-related differences in men and women, sex and interaction factors were also added to multivariable models. A logistic analysis was performed to test the effect of alcohol on indices of steatosis and stiffness.

## Results

The study cohort initially included 69 individuals; 62 of them completed the follow up and have been analysed here. A flowchart (*Suppl Table A*) shows causes of dropout. Thirty-six of them were non-drinker and 26 reported a mild alcohol consumption (AUDIT Score ≤ 7, and < 140 and 210 g/week alcohol consumption for women and men, respectively; the mean weekly alcohol consumption was 80 g/week in men and 60 in women). Their clinical characteristics at baseline are shown in Table 1. Participants had a mean age of 61 years; one third were women. They were obese, with a sub-optimal blood pressure control, Mean HbA1c of 56 mmol/mol and, as expected, slightly increased liver enzymes; however, phenotype, glucose control, lipid profile did not differ between the groups; the only significant difference emerged in sex, being females largely prevalent in the non-drinker subgroup. GFR was fully preserved in both groups, and most participants were normoalbuminuric. CAP, a quite reliable measure of liver steatosis, was slightly above the normal range, although not different between Alcohol- and Alcohol + subjects. Mean liver stiffness was normal in both groups (6.2 vs. 6.8 kPa, *p* = ns).

**Table 1:** Baseline clinical characteristics of the study cohort

Phenotype	Whole cohort (*n* = 62)	Alcohol - (*n* = 36)	Alcohol + (*n* = 26)	*p*
Age (years)	61.4 ± 11.3	60.2 ± 12.3	62.9 ± 9.6	0.357
Female (%)	20 (32)	16 (44)	4 (15)	**0.016**
T2D duration (years)	4.0 (0.5-7.0)	5 (1–9)	4 (0–7)	0.503
Weight (kg)	90.8 ± 18.1	87.8 ± 15.3	95.0 ± 21.0	0.124
BMI (kg/m^2^)	31.4 ± 5.0	31.1 ± 4.5	31.8 ± 5.7	0.563
Waist Circumference (cm)	111 ± 12	110 ± 10	113 ± 14	0.378
SBP (mmHg)	150 ± 19	146 ± 15	155 ± 24	0.101
DBP (mmHg)	82 ± 11	81 ± 10	83 ± 12	0.524
**Liver parameters**				
AST (IU/L)	34 ± 25	33 ± 25	35 ± 26	0.627
ALT (IU/L)	46 ± 41	43 ± 38	50 ± 45	0.585
GGT (IU/L)	74 ± 113	47 ± 34	61 ± 58	0.960
APh (IU/L)	76 ± 29	79 ± 33	72 ± 24	0.637
PLT (10^3/µl)	244 ± 68	242 ± 78	245 ± 46	0.824
Albumin (g/dl)	4.6 ± 0.3	4.6 ± 0.3	4.6 ± 0.4	0.625
Tot Bilirubin (mg/dl)	0.62 ± 0.32	0.56 ± 0.32	0.76 ± 0.11	0.098
PT (%)	99 ± 12	102 ± 10	92 ± 13	0.063
**Metabolic profile**				
Fasting glucose (mg/dl)	151 ± 54	154 ± 61	148 ± 44	0.934
HbA1c (mmol/mol)	56 ± 14	55 ± 13	57 ± 16	0.640
Insulin (µU/ml)	12.5 ± 8.8	11.2 ± 8.1	13.9 ± 9.5	0.327
Homa-IR	5.8 ± 3.8	5.1 ± 3.3	7.0 ± 4.5	0.176
Tot Cholesterol (mg/dl)	177 ± 43	177 ± 47	177 ± 38	0.968
HDL Cholesterol (mg/dl)	50 ± 14	52 ± 16	47 ± 11	0.235
LDL Cholesterol (mg/dl)	100 ± 38	101 ± 42	98 ± 32	0.767
Triglycerides (mg/dl)	166 ± 71	155 ± 62	180 ± 82	0.214
**Kidney function**				
Creatinine (mg/dl)	0.88 ± 0.27	0.87 ± 0.32	0.89 ± 0.18	0.745
eGFR (ml/min/1.73m^2^)	91 ± 18	91 ± 19	90 ± 16	0.770
ACR (mg/g)	6 (4–20)	6 (2–55)	5 (4–20)	0.369
Uric Acid (mg/dl)	5.2 ± 1.4	5.6 ± 1.4	4.6 ± 1.4	0.086
**Liver indices**				
CAP (dB/m, ref. >268)	333 (283–369)	321 (276–367)	349 (315–374)	0.126
LS (kPa, ref. >8)	6.4 (5.2–9.8)	6.2 (5.0-10.1)	6.8 (5.2–9.5)	0.916

During the six months of treatment with semaglutide, no variations in ongoing chronic (anti-hypertensive or hypolipidemic) therapies occurred. Alcohol + subjects did not report changes in their alcohol assumption (AUDIT score: 4 at T0 and T1). At the end of the follow-up, semaglutide showed a clinically relevant metabolic impact in both groups, improving the metabolic control (mean HbA1c reduction: -11 mmol/mol in Alcohol- and − 13 mmol/mol in Alcohol + subjects), determining a significant weight loss (a mean of -2.8 Kg in non-drinker and − 4.8 Kg in drinker subjects) and reducing liver enzymes. LDL cholesterol significantly improved (by 20%) only in Alcohol- subjects. CAP was significantly reduced in Alcohol + and LS in Alcohol- subjects (Table 2). A logistic analysis confirmed the absence of significant association between alcohol intake and deltas of CAP (*p* = 0.51) and LS (*p* = 0.88). Kidney function did not vary.

**Table 2:** Effect of semaglutide in the two study subgroups

	Alcohol - (*n* = 36)			Alcohol + (*n* = 26)		
	**T0**	**T1**	**p**	**T0**	**T1**	**p**
**Phenotype**						
Weight (kg)	86.5 ± 16.9	83.7 ± 13.8	**< 0.0001**	95.0 ± 21.0	90.2 ± 20.1	**< 0.0001**
BMI (kg/m2)	31.1 ± 4.5	29.6 ± 4.3	**< 0.0001**	31.8 ± 5.7	30.0 ± 5.6	**< 0.0001**
Waist Circumference (cm)	110 ± 10	105 ± 9	**< 0.0001**	113 ± 14	110 ± 13	**0.003**
PAS (mmHg)	146 ± 15	137 ± 15	**0.005**	155 ± 24	138 ± 15	**0.0005**
PAD (mmHg)	81 ± 10	78 ± 9	0.077	83 ± 12	76 ± 10	**0.010**
**Liver parameters**						
AST (IU/L)	33 ± 25	26 ± 11	0.126	35 ± 26	24 ± 12	**0.0003**
ALT (IU/L)	43 ± 38	26 ± 16	**0.014**	50 ± 45	30 ± 24	**< 0.0001**
GGT (IU/L)	83 ± 138	45 ± 61	**0.019**	61 ± 58	34 ± 27	**0.002**
APh (IU/L)	79 ± 32	84 ± 32	0.376	72 ± 24	70 ± 26	0.072
PLT (10^3/µl)	242 ± 78	244 ± 82	0.876	248 ± 46	215 ± 62	0.259
Albumin (g/dl)	4.6 ± 0.3	4.6 ± 0.3	0.752	4.6 ± 0.4	4.6 ± 0.2	0.514
Tot Bilirubin (mg/dl)	0.56 ± 0.32	0.57 ± 0.27	0.523	0.76 ± 0.31	0.68 ± 0.49	0.228
PT (%)	102 ± 10	102 ± 7	0.352	92 ± 13	88 ± 21	0.731
**Metabolic profile**						
Fasting Glucose (mg/dl)	154 ± 61	111 ± 19	**< 0.0001**	148 ± 44	114 ± 27	**< 0.0001**
HbA1c (mmol/mol)	55 ± 13	44 ± 7	**< 0.0001**	57 ± 16	44 ± 7	**< 0.0001**
Tot Cholesterol (mg/dl)	177 ± 47	160 ± 41	**0.013**	177 ± 38	149 ± 39	**0.005**
HDL Cholesterol (mg/dl)	52 ± 16	50 ± 16	0.212	47 ± 11	45 ± 9	0.282
LDL Cholesterol (mg/dl)	101 ± 42	80 ± 33	**0.014**	98 ± 32	77 ± 38	0.051
Triglycerides (mg/dl)	155 ± 62	150 ± 64	0.868	180 ± 82	132 ± 64	**0.0007**
**Kidney function**						
Creatinine (mg/dl)	0.87 ± 0.32	0.89 ± 0.41	0.934	0.89 ± 0.18	0.88 ± 0.20	0.861
eGFR (ml/min/1.73m^2^)	91 ± 19	91 ± 22	0.687	90 ± 16	91 ± 17	0.710
ACR (mg/g)	6 (2–55)	8 (4–14)	0.311	5 (4–20)	12 (4–17)	0.459
Uric Acid (mg/dl)	5.6 ± 1.4	5.7 ± 1.9	0.411	4.6 ± 1.4	5.1 ± 1.1	0.122
**Liver indices**						
CAP (dB/m)	321 (276–367)	297 (255–345)	0.126	349 (315–374)	313 (284–344)	**0.0005**
LS (kPa)	6.2 (5.0-10.1)	5.6 (4.6–7.2)	**0.006**	6.8 (5.2–9.5)	6.0 (4.8–8.1)	0.144

Circulating hormone levels at baseline and at the end of the treatment are reported in Table 3. After six-months of semaglutide, as expected, GLP-1 levels significantly - and similarly - raised in the two groups. Glucagon tended to be slightly reduced, and GIP slightly increased, in Alcohol + subjects.

**Table 3:** Effect of semaglutide on hormone levels in the whole cohort and in the two study subgroups

Whole cohort (*n* = 62)	T0	T1	*p*
Insulin, µU/ml	12.5 ± 8.8	12.9 ± 7.6	0.296
Glucagon, pg/ml	40.6 ± 15.4	37.4 ± 22.3	0.403
GLP-1, pM	84.8 ± 48.5	187.2 ± 57.1	**< 0.0001**
GIP, pg/ml	200.4 ± 185.4	192.1 ± 104.3	0.960
**Alcohol -** (*n* = 36)	**T0**	**T1**	**p**
Insulin, µU/ml	11.2 ± 8.1	11.7 ± 6.0	0.960
Glucagon, pg/ml	36.9 ± 16.3	38.2 ± 25.3	0.392
GLP-1, pM	76.4 ± 43.2	181.6 ± 55.6	**0.010**
GIP, pg/ml	241.1 ± 227.8	175.0 ± 120.4	0.190
**Alcohol +** (*n* = 26)	**T0**	**T1**	**p**
Insulin, µU/ml	13.9 ± 9.5	15.3 ± 10.2	0.151
Glucagon, pg/ml	45.3 ± 13.3	36.4 ± 18.5	0.779
GLP-1, pM	95.3 ± 54.5	193.9 ± 59.7	**0.002**
GIP, pg/ml	149.5 ± 100.4	212.8 ± 162.2	0.282

**Fig. 1:**
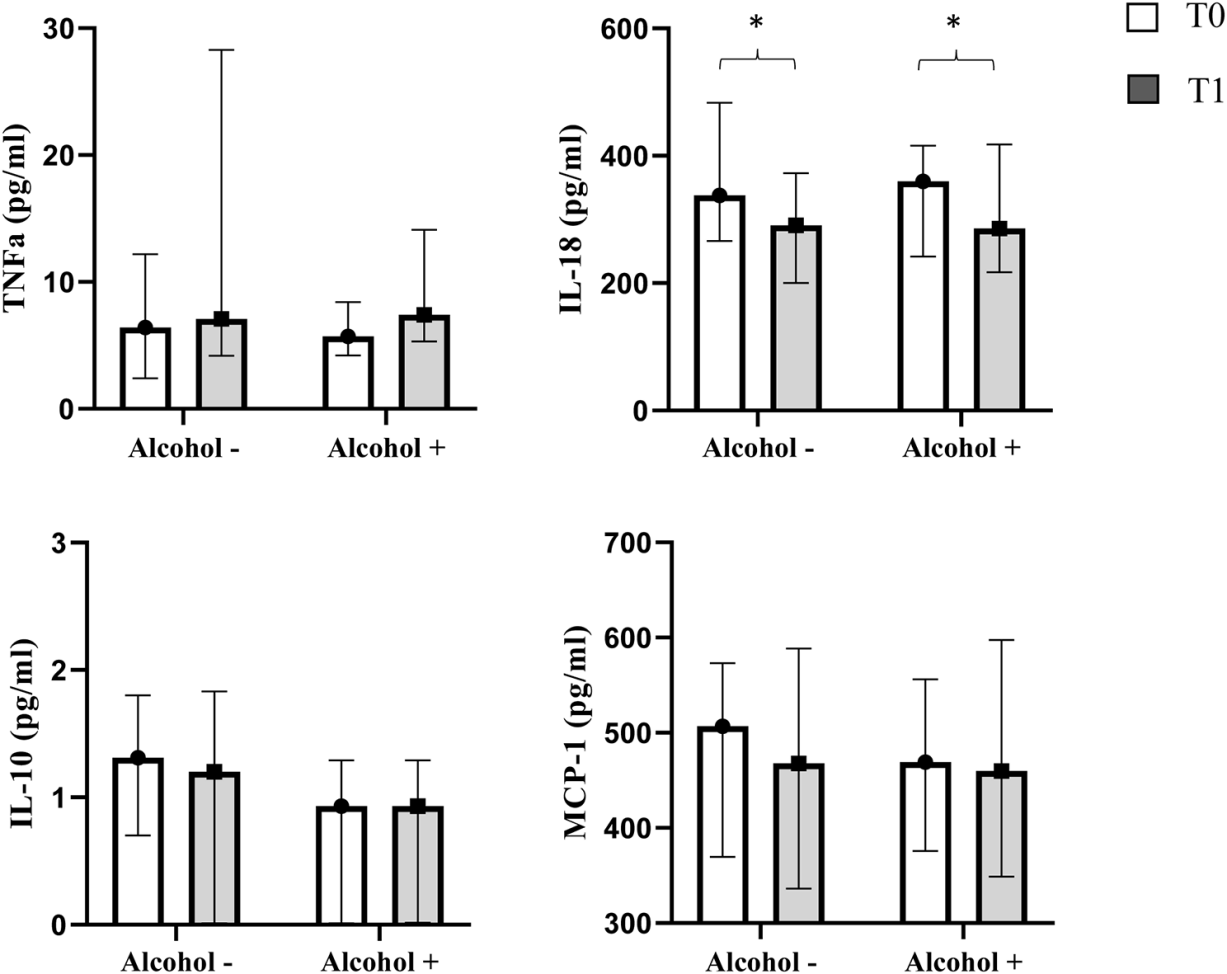
TNFα, MCP-1, IL-18, and IL-10 circulating levels at baseline and after treatment with semaglutide in the two study subgroups (Alcohol - and Alcohol +). Data are shown as median and IQ range. * *p* < 0.001

The cytokine pattern showed a similar behaviour in the two groups, with IL-18 significantly reduced by semaglutide treatment in the whole study cohort (from 353 [258–467] to 288 [212–381] pg/ml, *p* = 0.0002), and irrespective of alcohol consumption (Fig. 1).

At baseline, as expected, significant direct correlations emerged between CAP and LS and measures of adiposity (BMI and waist circumference), as well as liver enzymes; an interesting positive relation also linked glucagon with CAP (*r* = 0.4966, *p* = 0.0052). At T1, CAP reduction was related to weight loss (*r* = 0.385, *p* = 0.0041) and BMI reduction (*r* = 0.388, *p* = 0.0037), but not to glucose or HbA1c. CAP improvement was also related to GLP-1 increase (*r*=-0.415, *p* = 0.0251), with no difference between Alcohol + and Alcohol- individuals. No relations emerged between variations of inflammatory markers and metabolic or hepatic parameters. In a multivariate model including BMI, HbA1c, ALT, and GLP-1 variations after the treatment, CAP reduction remained associated only with GLP-1 rise (ß=-0.437, *p* = 0.0122); mild alcohol consumption did not change this effect.

## Discussion

This real-life study aimed at testing for the first time whether semaglutide would display a different efficacy in non-drinker and mild alcohol-consuming T2D individuals, paying special attention to metabolic effects, influence on the liver and anti-inflammatory action. The main results are: *(i)* the effect of semaglutide on glucose control, body weight and blood pressure values does not differ in the two subgroups, but non-drinker subjects encounter a significant reduction in LDL-cholesterol levels; *(ii)* the beneficial effects on liver enzymes are similar, and indices of liver steatosis and stiffness are reduced by semaglutide, LS more in Alcohol- and CAP more in Alcohol + subjects; *(iii)* the hormone profile does not significantly vary, except for the expected raise in GLP-1 levels; *(iv)* IL-18 levels are significantly reduced, irrespective of alcohol consumption.

Alcohol use is regarded as a main responsible for the development of liver metabolic abnormalities in people with [22] and without T2D [23, 24]. Alcohol consumers are characterized by a higher cardiovascular risk [25], with abstinence from alcohol able to improve the cardiovascular risk profile of these individuals [26]. In T2D, the influence of a mild alcohol consumption on the therapeutic effects of semaglutide has never been explored. Our data show that semaglutide similarly improves metabolic profile and reduces liver enzymes in Alcohol + and Alcohol- subjects, suggesting that a mild alcohol assumption does not interfere with semaglutide action. Differently to previous reports, alcohol consumers did not further reduce their alcohol assumption during the six-months of treatment with semaglutide, suggesting caution in extrapolating in the clinical practice results obtained in preclinical models [27, 28] or in heavy drinkers [29]. A reduction of LDL cholesterol following a short-term treatment with injective semaglutide in T2D subjects has been recently reported [30]; our result of a significant reduction of LDL-cholesterol levels following semaglutide treatment only in non-drinkers, in the absence of any variation on chronic therapies over the six months of treatment, deserves to be confirmed in larger studies.

Metabolic syndrome increases the risk of liver fibrosis in alcohol consumers [31], and it is known that low dose semaglutide injections improve ALT and radiologic features in MAFLD [32]. Our observation of a significant reduction of liver stiffness in non-drinker T2D subjects is novel, and likely indicates a reinforced liver protection of GLP-1 receptor agonists in non-alcohol consumers. The key role of weight loss in improving CAP [33, 34], an indicator of liver fat content is confirmed.

In our study cohort, insulin, glucagon and GIP plasma concentrations did not vary after semaglutide, reinforcing the concept that metabolic efficacy of this drug is mediated by clinical effects, rather than circulating levels of these hormones. We should also consider with caution the observed raise in GLP-1 levels, likely due to the large (94%) structural homology existing between native human GLP-1 and semaglutide, that induces to think that what we have measured is largely accounted by circulating levels of the drug, that shows a much long half-life (> 160 h). To our knowledge, no ELISA kit able to distinguish between GLP-1 and semaglutide has been so far developed. However, such parameter shows an intrinsic relevant clinical efficacy, being the strongest determinant of CAP reduction in the multivariate analysis, and not being influenced by presence of absence of alcohol consumption.

Semaglutide has proven anti-inflammatory effects in animal models of obesity and metabolic abnormalities [35–37]; no information is, so far, available in human beings, although a randomized study dealing with such issue has been recently started [38]. Another innovative aspect of the present study is to evaluate for the first time the effect of semaglutide on circulating pro-inflammatory cytokines and chemokines. Semaglutide does not modify TNFα, MCP-1 and IL-10 plasma levels, while a significant reduction of IL-18 was observed in both non-drinker and alcohol consumer T2D subjects. IL-18-mediated diseases involve multiple organs and systems, particularly systemic hyperinflammation, which is consistent with the pleiotropic amplification of several pathways, including INFγ; IL-18 signaling controls energy homeostasis, pancreatic islet immunity and liver integrity during nutritional stress [39]. At the same time, clinical observations implicate IL-18 in various metabolic diseases including obesity, T2D and MAFLD [40–42]. The significant reduction of IL-18 systemic levels is likely to participate in the anti-inflammatory effects exerted by semaglutide on the liver.

## Conclusion

We show here as, in T2D subjects, semaglutide exerts its metabolic benefits, including a reduction of liver fat accumulation and stiffness, similar in non-drinker subjects and in mildly alcohol consumers; a clear reduction of IL-18 is reported for the first time in human beings.

We should acknowledge some limitations of our study, mainly residing in the relatively small size of the cohort, the prevalence of females among non-drinker subjects, the use of surrogate markers of steatosis and fibrosis (CAP and LS) rather than more sophisticated instrumental approaches, like magnetic resonance, in detecting liver improvement induced by semaglutide.

### Electronic supplementary material

Below is the link to the electronic supplementary material.

Supplementary Material 1

## Data Availability

The study dataset is available from the corresponding author upon reasonable request.
