# Supplementary material for: Response to semaglutide of non-drinker subjects with type 2 diabetes
Source: Diabetol Metab Syndr. 2024 May 17;16:103. doi: 10.1186/s13098-024-01344-6 (PMC11100230; doi:10.1186/s13098-024-01344-6)
Supplement: Supplementary file 1 — Supplementary Material 1 [file 13098_2024_1344_MOESM1_ESM.docx]

**Suppl Figure A Study flowchart**.

Total number of eligible patients*

N=95

Excluded 26

(20 preferred oral semaglutide,

6 refused the treatment)

69 enrolled

Excluded 7

(4 lost at the follow up,

3 did not tolerate semaglutide 1 mg)

62 completed the study

*consecutively referring to our outpatient clinic between January and December 2023, matching inclusion criteria and deserving semaglutide treatment
